# Recruitment of aged donor heart with pharmacological stress echo. A case report

**DOI:** 10.1186/1476-7120-4-3

**Published:** 2006-01-24

**Authors:** Giorgio Arpesella, Sonia Gherardi, Tonino Bombardini, Eugenio Picano

**Affiliations:** 1 Department of Surgery and Transplants, University of Bologna, Italy; 2 Operative Cardiologic Unit, M. Bufalini Hospital, Cesena, Italy; 3 Department of Echocardiography, Institute of Clinical Physiology, National Council of Research, Pisa, Italy

## Abstract

**Background:**

The heart transplant is a treatment of the heart failure, which is not responding to medications, and its efficiency is already proved: unfortunately, organ donation is a limiting step of this life-saving procedure.

To counteract heart donor shortage, we should screen aged potential donor hearts for initial cardiomyopathy and functionally significant coronary artery disease.

Donors with a history of cardiac disease are generally excluded. Coronary angiography is recommended for most male donors older than 45 years and female donors older than 50 years to evaluate coronary artery stenoses. A simpler way to screen aged potential donor hearts for initial cardiomyopathy and functionally significant coronary artery disease should be stress echocardiography.

**Case report:**

A marginal donor (A 57 year old woman meeting legal requirements for brain death) underwent a transesophageal (TE) Dipyridamole stress echo (6 minutes accelerated protocol) to rule out moderate or severe heart and coronary artery disease. Wall motion was normal at baseline and at peak stress (WMSI = 1 at baseline and peak stress, without signs of stress inducible ischemia). The pressure/volume ratio was 9.6 mmHg/ml/m^2 ^at baseline, increasing to 14 mmHg/ml/m^2 ^at peak stress, demonstrating absence of latent myocardial dysfunction.

The marginal donor heart was transplanted to a recipient "marginal" for co-morbidity ( a 63 year old man with multiple myeloma and cardiac amyloidosis , chronic severe heart failure, NYHA class IV).

Postoperative treatment and early immunosuppressant regimen were performed according to standard protocols.

The transplanted heart was assessed normal for dimensions and ventricular function at transthoracic (TT) echocardiography on post-transplant day 7.

Coronary artery disease was ruled out at coronary angiography one month after transplant; left ventriculography showed normal global and segmental LV function of the transplanted heart.

**Conclusion:**

For the first time stress echo was successfully used in the critical theater of screening potential donor hearts. This method is enormously more feasible, less expensive, and more environmentally sustainable than any possible alternative strategy based on stress scintigraphy perfusion imaging or coronary angiography. The selection of hearts "too good to die" on the basis of bedside resting and stress echo can be a critical way to solve the mismatch between donor need and supply.

## Background

The heart transplant is a treatment of the heart failure, which is not responding to medications, and its efficiency is already proved: unfortunately, organ donation is a limiting step of this life-saving procedure.

In fact, the selection of the organs that will be transplanted is based on two factors: a suitable donor, without systemic diseases or neoplasia, active bacterial or viral infections, or anyway according to Health Guidelines. For the organ "heart", an additional criterion is represented by the age; this factor is not so important in and by itself, but for the associated age-dependent risk for asymptomatic coronary artery disease or latent cardiomyopathy [1]. Generally, the age threshold is 55 years, older donors being accepted in special cases, when the recipient's condition is very poor and represents an emergency [2].

Heart donor shortage is a society problem. Patients in heart transplant waiting list have a 7.3% death rate, and the average waiting time is 2 to 3 years. In Italy, about 650 patients are in transplant list and only about 300 transplant are performed each year, with a plateau in the last 5 years.

Furthermore, there is a large amount of marginal recipients, both for advanced age (> 65 years) or co-morbidity in younger recipients [3,4].

**Figure 1:**
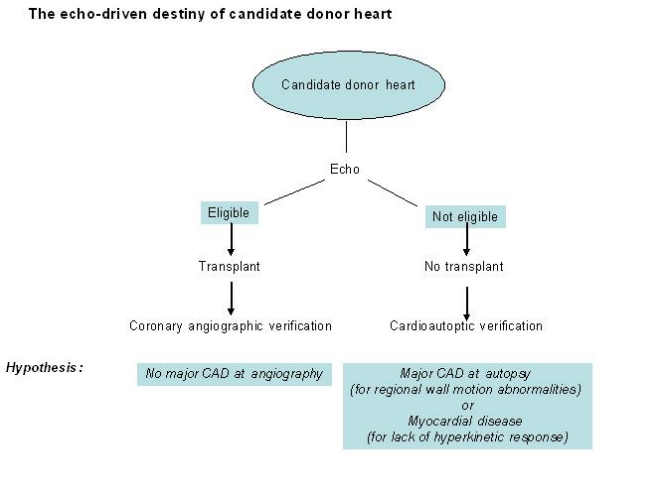
The echo-driven destiny of candidate donor heart. To screen aged potential donor hearts for initial cardiomyopathy and functionally significant coronary artery disease, potential donors aged > 55 years will undergo pharmacological stress echo (with either dipyridamole or dobutamine). Hearts with normal regional and global left ventricular function will be recruited. Hearts with stress echo positivity will be excluded. Excluded hearts will undergo cardioautoptic verification to challenge stress echo results versus a pathologic gold standard. Included hearts will be transplanted and the stress echo result will be challenged versus clinical outcome and angiographic verification 1 month after transplant. The Adonhers (Aged Donor Heart Rescue by Stress Echo) Project Additional files (video clips) 1, 2, 3, and 4. A transesophageal pharmacological stress echo test was performed, following the protocol of American Society of Echocardiography [5], using: Dipyridamole (0.84 mg/kg in 6'). The echocardiographic study is divided into two different parts: 1 Segmental wall motion- it is the essential step for the evaluation. The donor with abnormal wall motion at baseline or during stress will be excluded from the donor ship. The donor showed normal regional wall motion at baseline and during stress. (WMSI = 1 at baseline, intermediate and peak stress). Global and regional wall motion is shown in 2 and 4 chambers view at baseline (Additional files 1 and 2) and at peak stress (Additional files 3 and 4). 2 The force-frequency relation (FFR). During the procedure, pressure and ECG were recorded every minute. Brachial blood pressure was measured with cuff sphygmomanometer. In every phase of the stress echo, the projections of the 4 chamber and of the apical 2 chambers were recorded to calculate the left ventricular telesystolic volume. This allowed us to calculate the systolic pressure/left ventricle telesystolic volume ratio in every moment when increasing the cardiac frequency during the stress and building, off-line, the force-frequency relation of the left ventricle. The donor had normal response at rest and with normal regional wall motion during pharmacological stress echo and was considered suitable donor; indeed, this response pattern proved a good coronary and functional reserve of the heart. (Images from Sonia Gherardi, Director Echo lab, Cardiology Division, Cesena Hospital, Italy)

To counteract heart donor shortage, we should screen aged potential donor hearts for initial cardiomyopathy and functionally significant coronary artery disease: a way to select hearts "too good to die" on the basis of bedside resting and stress echo can be a critical way to solve the mismatch between donor need and supply. The Adonhers (Aged Donor Heart Rescue by Stress Echo) Project was approved by the Ethical Committee of the Sant'Orsola Hospital of Bologna in January 2005 (Figure 1). The project is now active in the Emilia-Romagna Region in its pilot phase.

## Case report

### The marginal donor

A 57 year-old woman had a traffic accident with traumatic brain death and was selected for organ donation. The woman was older than the upper age limit of 55 for heart donation.

Next, a careful cardiac history took form, with no background data of cardiac symptoms at rest or under effort. There was a history of mild systemic hypertension. A 12 -lead electrocardiogram resulted in non-specific T wave changes, often seen in brain death.

### Standard TT echocardiography

At time when doses of intravenous inotropic agents have been lowered to as low as is compatible with adequate blood pressure and cardiac output (noradrenaline 0.13 γ/Kg/min + dobutamine 2.8 γ/Kg/min) and after adequate fluid infusion a TT echocardiogram was taken demonstrating normal global and segmental ventricular function, no valvular disease [5].

### The pharmacological stress echo

To rule out moderate or severe heart and coronary disease, a TE Dipyridamole stress echo (6 minutes accelerated protocol) was performed [6].

The echo images were tape-recorded and periodically digitized. During the procedure, pressure and ECG were recorded every minute [7]. Results of the transesophageal dipyridamole stress echo are shown in Additional Files (video clips) 1, 2,3, and 4. There was a normal response of the left ventricle during stress. Brachial blood pressure was measured with cuff sphygmomanometer. In every phase of the stress echo, the projections of the 4 chambers and of the apical 2 chambers were recorded to calculate the left ventricular telesystolic volume. This allowed us to calculate the systolic pressure/left ventricle telesystolic volume ratio in every moment when increasing the cardiac frequency during the stress and building, off-line, the force-frequency relation (FFR) of the left ventricle [8].

### The results of the stress

Wall motion was normal at baseline and at peak stress (WMSI = 1 at baseline and peak stress), without signs of stress inducible ischemia (Additional files 1, 2, 3, and 4).

**Figure 2:**
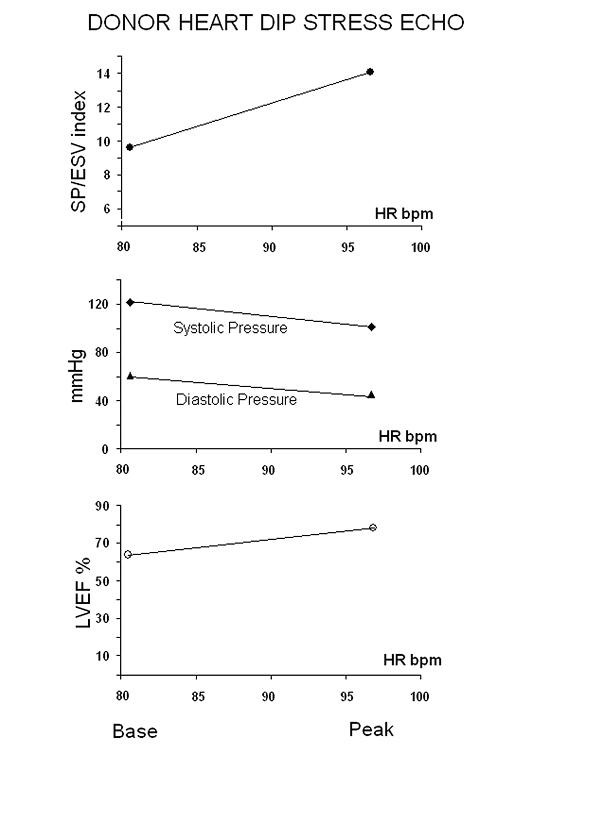
The force-frequency curve with DIP stress echo in the Donor heart. Upper panel: An increased heart rate is accompanied by smaller end-systolic volumes (normal up sloping FFR). The pressure/volume relation was 9.6 mmHg/ml/m^2 ^at baseline, increasing to 14 mmHg/ml/m^2 ^at peak stress, FFR slope = 28 mmHg/ml/m^2^/bpm demonstrating absence of latent myocardial dysfunction. Middle and lower panels. Systemic blood pressure and LVEF % at baseline and peak stress. A stress induced increase in LVEF simultaneously to stress induced systolic and diastolic pressure decrease is confounding for the contractile reserve evaluation.

The pressure/volume relation was 9.6 mmHg/ml/m^2 ^at baseline, increasing to 14 mmHg/ml/m^2 ^at peak stress, FFR slope = 28 mmHg/ml/m^2 ^/bpm demonstrating absence of latent myocardial dysfunction (Figure 2).

Systemic blood pressure decreased from 120/60 mmHg at baseline to 100/45 mmHg at peak stress.

LVEF % increased from 63% at baseline to 77% at peak stress.

Based on the dipyridamole stress results, despite the age beyond the 55 years limit, the heart was chosen for orthotopic heart transplantation, and was explanted with standard technique.

### The marginal recipient

**Figure 3:**
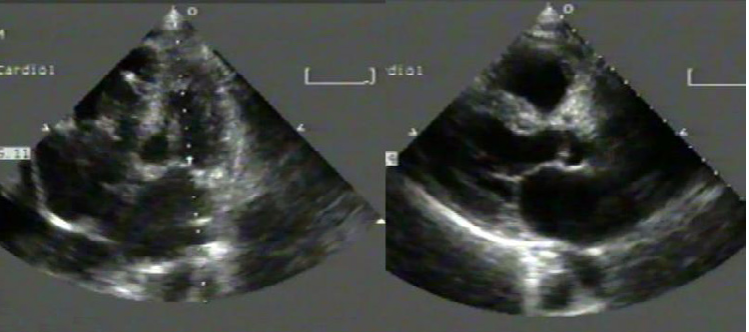
Two-dimensional transthoracic echocardiogram recorded in the marginal recipient with cardiac amyloid. In both the parasternal long-axis (right) and apical four-chamber (left) images note the homogeneous echo intensity of the myocardium, which is noted as a bright "speckling" appearance. Both the mitral and aortic valves are also thickened. The patient had severe ventricular dysfunction and advanced heart failure symptoms (NYHA Class IV). (Images from Marinella Ferlito, Director Echo lab, and Prof Claudio Rapezzi, University Cardiology Division, S. Orsola Hospital, Bologna, Italy) Additional files (video clips) 5 and 6. Two-dimensional transthoracic echocardiogram of the donor heart grafted in the marginal recipient. In both the parasternal long-axis (Additional file 5) and apical four-chamber (Additional file 6) views note the excellent global and regional ventricular function. Chambers and cardiac structures are normals. (Images from Marinella Ferlito, Director Echo lab, and Prof Claudio Rapezzi, University Cardiology Division, S. Orsola Hospital, Bologna, Italy)

The recipient was "marginal" for co-morbidity. He was a 63 year old man with multiple myeloma and cardiac amyloidosis, chronic severe heart failure, NYHA class IV, LVEF = 13% (TT echocardiography, Figure 3) [9].

### The heart transplant

The operation was performed by way of a median sternotomy incision, with cannulation of the aorta and both venae cavae. The implantation procedure required one hour. The incision was closed after placement of temporary pacing wires and chest drainage catheters [10].

### The transplant outcome

After the transplant, the patient underwent a routine treatment and follow-up procedure.

Standard postoperative treatment was performed, mobilization and physical therapy was begun the day 7 after surgery.

Early immunosuppressant regimen was started according to the standard protocols.

### The echo control

The transplanted heart was assessed normal for dimensions and ventricular function at TT echocardiography on post-transplant day 7 (Additional files 5 and 6).

### The transplanted heart coronary angiography

**Figure 4:**
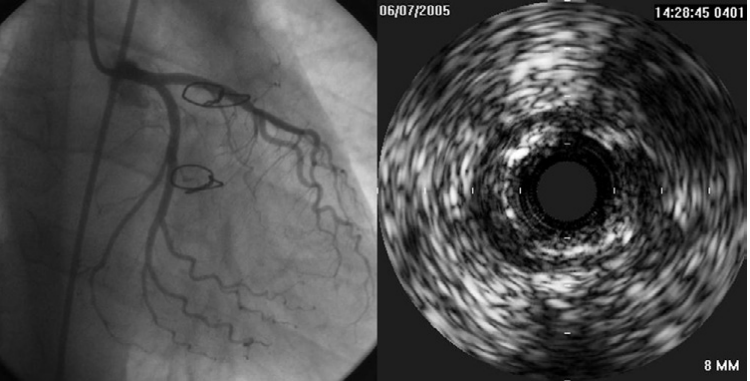
Graft coronary artery imaging- appearance by angiography and intravascular ultrasound 1 month after follow-up. Excellent angiographic result demonstrating concordance with the pre-transplant potential donor stress echo (no inducible ischemia). The left panel shows angiogram obtained on the transplanted heart 1 month after engraftment. Angiographic imaging of the midportion of the LAD only shows minor lumen irregularities within the vessel. The right panel shows intravascular ultrasound images obtained in the same coronary segment. (images from Prof Claudio Rapezzi and Dr Carlo Magelli, responsible of the Clinical Heart Transplant Program, University Cardiology Division, S. Orsola Hospital, Bologna, Italy)

Coronary artery disease of the transplanted heart was ruled out at coronary angiography one month after transplant; left ventriculography showed normal global and segmental LV function (Figure 4).

## Discussion

Supply of donor hearts is a critical rate-limiting step in heart transplantation. An effective way to solve the current shortage would be to accept an upward shift of the age cutoff limit (from current 45 to 70 years) but age-related high prevalence of asymptomatic coronary artery disease and occult cardiomyopathy severely limit the feasibility of this approach.

Pharmacological stress echo is inexpensive, non-invasive and allows a simultaneous evaluation of inducible ischemia and contractile reserve of the left ventricle – therefore, it is capable to unmask prognostically meaningful occult coronary artery disease or cardiomyopathy [6,7].

The excellent prognosis associated with a negative stress echo test is already well documented by databases of thousands of patients with known or suspected coronary artery disease [11].

Force-Frequency relationship (FFR) is a theoretically and methodologically robust method for the assessment of left ventricular contractility and can be assessed non-invasively during exercise echo. [8] Previous studies demonstrated that an up sloping FFR better identifies hearts without latent left ventricular dysfunction than standard 5% of cutoff stress increase in LVEF [12].

This first described case regards stress in special conditions – such as those occurring in a candidate donor. Drug infusion may create special problems of hemodynamic instability (and, therefore, of stress tolerance) and echocardiographic feasibility and interpretation of the results.

For this reason, we considered ethically sound to use the organ considered suitable using this method, for transplant in recipients in emergency or suboptimal conditions. It is already a current policy to transplant suitable organs removed from donors aged more than 55 years, to patients with relative contraindications to transplant. In this context, a stress echo test will be a further reason of warranty for the recipient, while a change in policy based on stress echo results would only be unwarranted at this stage of evidence. It is obvious that the ultimate target may be the removal of the "age factor" and its replacement with a stress echo driven selection, but this far-reaching change in selection policy has to go through several intermediate check points.

## Conclusion

The relevance of this case is:

*Scientific*: for the first time, a largely validated technology such as stress echo is used in the critical theater of screening potential donor hearts.

*Clinical*: the efficacy of an echo-driven strategy for selecting donor heart is enormously more feasible, less expensive, and more logistically sustainable than any possible alternative strategy based on stress scintigraphy perfusion imaging or coronary angiography, which cost respectively 3 times and 10 times more than a stress echo and are much more logistically demanding.

*Social*: heart donor shortage is a society problem. Patients in heart transplant waiting list have a 7.3% death rate, and the average waiting time is 2 to 3 years. In Italy, about 650 patients are in transplant list and only about 300 transplants are performed each year, with a plateau in the last 5 years. A better way to select hearts "too good to die" on the basis of bedside resting and stress echo can be a critical way to solve the mismatch between donor need and supply.

*Economic*: the cost of a donor heart is estimated around 200.000 € in the "transplant black market". We can recruit otherwise ineligible donor at the cost of 1 stress echo, with enormous obvious downstream economic benefits.

## Competing interests

The author(s) declare that they have no competing interests.

## Supplementary Material

Additional file 1 Click here for file

Additional file 2 Click here for file

Additional file 3 Click here for file

Additional file 4 Click here for file

Additional file 5 Click here for file

Additional file 6 Click here for file
